# Donor-Site Morbidity in Anterior Cruciate Ligament (ACL) Reconstruction With All-Soft Tissue Quadriceps Tendon Autograft vs. Hamstring Tendon Autograft: A Retrospective Monocentric Observational Study

**DOI:** 10.1155/aort/8833546

**Published:** 2025-05-19

**Authors:** Simone Giusti, Marco Susca, Simona Cerulli, Edoardo De Fenu, Ezio Adriani

**Affiliations:** Complex Operational Unit of Sports Traumatology and Joint Reconstruction, Fondazione Policlinico Universitario Agostino Gemelli IRCCS, Rome, Italy

## Abstract

**Background:** Graft choice, together with operative technique, remains the most controversial topic surrounding ACL reconstruction. The ideal graft choice should recreate normal anatomy and biomechanics, allow for rapid return to play and have minimal harvest-site morbidity. The purposes of this study were to compare donor-site morbidity in all-soft-tissue quadriceps autograft vs. hamstring autografts based on Hacken et al.'s ACL Donor-Site Morbidity Questionnaire (32,587,874) and to assess the role played by external factors such as sex, mood, activity level and smoking status.

**Materials and Methods:** We performed a retrospective analysis of our patients' records to identify individuals who were 30 years old or younger at the time of surgery and underwent ACL reconstruction using the anteromedial portal technique, without any additional treatments for ligament or meniscal injuries. At 12 months postintervention, donor-site morbidity was evaluated using the ACL donor-site morbidity questionnaire by Hacken et al. (2020). Analyses were performed using Jamovi freeware Version 2.3.19.0 (the Jamovi project, 2021). Independent samples t-test with Cohen's d as the effects' size statistics were used to compare donor-site morbidity and functional outcomes.

**Results:** Significant differences between quadriceps tendon (QT) and STG groups were found for ACL donor-site morbidity questionnaire total score, numbness, size of numbness and muscle atrophy, all in favour of the QT cohort. Weak associations were found between female sex and low mood, both negatively impacting the reported donor site morbidity. No statistically significant differences were found for functional outcomes.

**Conclusion:** ACL reconstruction with all-soft-tissue QT autograft showed overall superior donor-site morbidity outcomes when compared with HT autograft. Statistically significant results were also found in favour of QT when comparing numbness and size of numbness at the donor site and self-perceived muscle atrophy. Female sex and low mood have been found to impact donor-site morbidity negatively although larger samples are necessary to confirm this association. Graft choice in ACL reconstruction should always remain an individualized choice, but QT should be considered an equal, if not superior, alternative to other autologous autografts when comparing donor-site morbidity.

**Trial Registration:** CINECA: 6458

## 1. Introduction

Anterior cruciate ligament (ACL) rupture affects 120,000 people per year in the United States alone. These injuries are extremely common amongst young, active patients, with a peak incidence in professional female athletes [1]. The main consequences of ACL injuries are attributed to altered knee kinematics and joint laxity, which ultimately result in knee instability, especially during exercise [2–4]. Reconstructing the ACL promptly is of vital importance in the younger patient population. This is crucial to avoid meniscal tears [5] as well as mid- and long-term consequences of joint instability such as chondral degeneration and early-onset knee osteoarthritis. Graft choice, together with operative technique, remains the most controversial topic surrounding ACL reconstruction [6]. The ideal graft choice should recreate normal anatomy and biomechanics, allow for rapid return to play (RTP) and have minimal harvest-site morbidity. However, the optimal graft choice is often considered to be the one with which the surgeon is most experienced, the most common choices being autologous grafts between bone–patellar tendon–bone (BPTB), hamstring tendons (STG) and quadriceps tendon (QT). The use of allografts has recently fallen out of choice due to issues with long-term reliability, especially in primary ACL reconstructions and highly active young patients [7]. One of the main factors to consider when choosing the best patient-specific graft is donor-site morbidity. ACL reconstruction with QT autograft has been associated with lower donor-site morbidity due to shorter skin incisions resulting in lower regional hypoesthesia, pain and irritation [8–11]. QT also appears to be associated with decreased risks of infection and injury to the infrasaphenous branch [12]. With regards to patient-reported outcome measures (PROMs), postoperative functional outcomes, rerupture rates and postoperative laxity measures, all available data appear similar amongst the three graft choices.

Patient age appears to be a crucial aspect in graft choice, with QT preference growing rapidly in the young and very young patient demographic [13]. QT graft seems to be used primarily by more experienced surgeons [14]; however, with new harvesting tools and fixation devices, there seems to be a positive trend where the choice of the graft is becoming more accessible. The purposes of this study were (1) to compare donor-site morbidity based on Hacken et al.'s ACL Donor-Site Morbidity Questionnaire in young patients (≤ 30 years old) who underwent surgical ACL reconstruction with QT autograft and STG autograft and (2) to assess the relationship between external factors such as sex, mood and smoking habit and donor-site morbidity. Our hypothesis was that the donor-site morbidity between the two grafts would be similar in the long term with, perhaps, higher pain scores in the QT group in the initial postoperative phase.

## 2. Methods

### 2.1. Study Sample

The study was conducted at Fondazione Policlinico Universitario Agostino Gemelli IRCCS University Hospital, Rome, Italy.

We performed a retrospective analysis of our patients' records to identify individuals who were 30 years old or younger at the time of surgery. Patients under the age of 16 were purposely excluded as physeal-sparing techniques were often necessary in these cases. The patients we identified underwent ACL reconstruction surgery using the anteromedial portal technique, without any additional treatments for ligament (ALL or LET tenodesis) or meniscal injuries. All patients received femoral fixation with Ultrabutton (Smith&Nephew, London, UK) and tibial fixation with Biosure Regenesorb interference screws (Smith&Nephew, London, UK). The study period spanned from 1 January 2022 to 30 December 2022, and we included only those patients who had a minimum follow-up period of 12 months. Written informed consent was provided from the patients. Institutional review board and ethical committee approval was obtained before the initiation of data collection.

### 2.2. Baseline Evaluation

Personal and clinical data including age, height, weight, foot dominance (defined as preferred kicking limb), smoking habits and activity levels were obtained at the preoperatory visit. Patients were asked to provide self-reported information on any occurrence of ACL injury at any point in their life, as well as any hamstring strain injury resulting in time loss (missing at least one game of competitive sport) during the past 12 months. Body weight and height were assessed using an analogue column scale with a built-in stadiometer. The body mass index (BMI) calculated as body weight (kg) divided by the square of height (m^2^). Activity level was evaluated through clinical interview using the Tegner Activity Scale (TAS) (4,028,566). The TAS scale measures an individual's level of activity on a scale from 0 to 10. A score of 0 indicates either being on sick leave or having a disability, while a score of 10 represents involvement in competitive sports, such as soccer, at a national or worldwide top level. Regular engagement in leisure or competitive sports is necessary to attain activity levels 6–10 (4,028,566). All patients had also been asked to complete a Patient Health Questionnaire (PHQ-9) on the day of the procedure to assess their mood.

### 2.3. Surgery

The ACL reconstruction operation was performed on all patients utilizing either autologous hamstring autograft (HT) or all-soft-tissue QT autograft. All the procedures were conducted by the same senior surgeon (EA). A single suction drain was placed in the antero-lateral portal in all procedures and removed day 1 postreconstruction. The ACL reconstruction surgery was performed on all patients within a time frame of 30 days after the first injury (range: 3–28 days). Average intraoperative time was 38 min (range: 32–49 min) whereas mean tourniquet time was 32 min (range: 27–36 min).

### 2.4. Follow-Up Visit

Reruptures or the occurrence of other injuries, donor-site morbidity, self-reported RTP and functional outcomes were assessed at a follow-up visit performed 12 months after the surgical procedure.

Donor-site morbidity was evaluated using the ACL donor-site morbidity questionnaire by Hacken et al. (32,587,874). Every question offered four potential answers that corresponded to escalating symptom intensity and patient dissatisfaction. Answer options were assigned a score ranging from 0 to 10 based on the degree of severity or functional limitation. The highest attainable score was 100, signifying a complete absence of complaints. The total scores were split into four groups that describe the overall morbidity following surgery: excellent (93.3 points), good (80.0–93.2 points), fair (50.0–79.9 points) and poor (49.9 points). All data are available upon request from the authors.

## 3. Results and Statistical Analysis

Analyses were performed using Jamovi freeware Version 2.3.19.0 (the Jamovi project, 2021).

A total of 87 patients were identified through record searching. Four patients were lost to follow-up; therefore, a total of 83 patients were included in the study. Mean age at time of surgery was 23.8 old with ranges 18–30. Sex distribution saw 51 males (61.4%) and 32 females (38.6%). Mean BMI was 23.9 ± 2.8 kg/m^2^ with BMI ranging from [15] 5–34.6. Average TAS was 7.7 ± 1.3 points. No intraoperative complications were reported, and all patients were discharged at home on the first postoperative day.  Table 1 summarises participant demographics. Overall, 46 (55.4%) patients received QT and 37 (44.6%) received HT, with the majority of the QT population being male (76.1%) and the majority of the HT population being female (56.8%). Patient characteristics shown with graft type are available in Table 2. Graft selection choice was not casual and down to surgical preference of the senior operating surgeon. At 12 months, rerupture rate was 0%.

**Figure 1:**
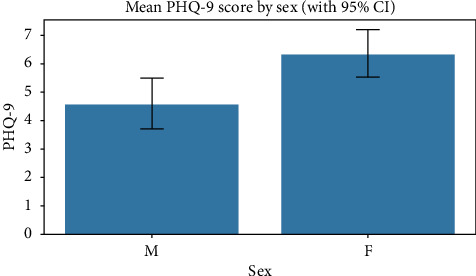
Mean PHQ-9 score by sex.

A Whitney *U* test was conducted to compare PHQ-9 scores between sexes, yielding a statistically significant difference (*p* 0.0016) with a small to moderate effect size (*r* 0.3472). Female participants (32) demonstrated higher PHQ-9 scores with a mean of 6.31 (SD 2.48) compared with male participants (51) who had a mean score of 4.55 (SD 3.16), representing a mean difference of 1.76 points on the PHQ-9 scale (Figure 1). This finding suggests that female patient reported significantly higher levels of depressive symptoms compared with their male counterparts, with the moderate effect size indicating a clinically meaningful difference.

On the contrary, we conducted a Spearman correlation analysis which indicated no significant linear relationship between age and depression severity.

**Figure 2:**
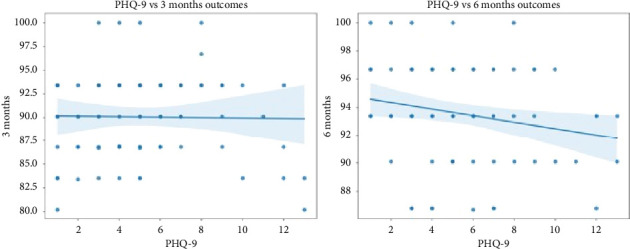
Mean PHQ-9 score vs. donor-site morbidity at 3 and 6 months.

We then examined the relationship between PHQ-9 scores and overall donor-site morbidity outcomes as measured using the Hacken questionnaire at 3 and 6 months (Figure 2). For the 3-month outcome, the Pearson correlation coefficient was found to be −0.017, indicating almost no linear association. The Spearman correlation was 0.079 with a *p* value of 0.478, also reflecting a nonsignificant monotonic relationship. In the case of the 6-month outcome, the Pearson correlation coefficient was slightly higher at −0.206, hinting at a weak negative relationship, while the Spearman correlation was −0.205 with a *p* value of 0.063, which is marginally above the conventional 0.05 significance threshold. Overall, these results indicate that at 6 months, depression severity may be associated with increased donor site morbidity, with no meaningful association at the 3-month mark.

Interestingly, when analysing the relationship between sex and overall donor-site morbidity using the Mann–Whitney *U* test, there was not statistically significant association at the 3-month mark.

This changed at 6 months where our analysis yielded a *p* value of 0.0405, which is statistically significant at the conventional 0.05 level. The effect size for the 6-month comparison was *r* 0.2249, suggesting a small difference, where females had a mean score of 92.57 (SD 3.66) compared with males' 94.24 (SD 3.15). These results collectively suggest that while donor-site scores did not differ markedly by sex at the 3-month follow-up, a statistically significant divergence emerged at the 6-month mark, with males exhibiting slightly higher scores than females.

Female sex was also associated with higher Visual Analogue Scale (VAS) scores at the 7-day postoperative mark (*p* = 0.03) whereas the relation between the two variables was not significant at 1 and 14 days postintervention, suggesting that while the early postintervention period does not exhibit marked sex differences in pain perception, females tend to experience higher pain levels at 1 week. The trend converges by Day 14.

No statistically significant relationship was found between age and donor-site morbidity at either the 3- or 6-month mark. Similar results were found for the association between smoking status and donor-site morbidity and between Tegner activity scores and donor-site morbidity.

A comprehensive statistical analysis was conducted to examine the differences in donor-site morbidity between QT (46) and HT (37) groups at both 3- and 6-month time points using the Kruskal–Wallis test. At 3 months postintervention, no statistically significant differences were observed between the groups (*H* = 0.1489 and *p* = 0.6996) with the HT group showing a mean score of 89.92 (SD: 4.92) and the QT group displaying a similar mean score of 90.17 (SD: 4.03). However, at the 6-month follow-up, significant differences emerged between the groups (*H* = 5.8964 and *p* = 0.0152), with the QT group demonstrating significantly higher scores (*M* = 94.48 and SD: 2.96) compared with the HT group (*M* = 92.51 and SD: 3.71). The effect size increased substantially from 0.0018 at 3 months to 0.0719 at 6 months, indicating a strengthening relationship between surgical technique and donor-site scores over time. The smaller standard deviation in the QT group at 6 months (2.96 vs. 3.71) also suggests more consistent outcomes with QT autografts.

The relationship between the graft type and VAS pain scores at days 1, 7 and 14 days postintervention was also examined using the Mann–Whitney *U* tests. At Day 1, there was no statistically significant difference between the groups (*p =* 0.3433). By Day 7, a nonsignificant difference emerged (*p* 0.0695), with the QT group showing higher pain scores (M = 3.24 and SD: 1.21) compared with the HT group (M = 2.81 and SD: 1.13). At Day 14, the difference between groups was not statistically significant (*p =* 0.3139).

Lastly, we analysed the relationship between HT and QT groups and singular items which make up the Hacken Questionnaire. A statistically significant difference in numbness outcomes (*p*  = 0.00007) was found, with patients who received QT reporting significantly better outcomes (M = 9.57 and SD: 1.12) compared with HT patients (M = 8.22 and SD: 1.67). The difference was also statistically significant in favour of QT when looking at the size of the numbness QT (M = 9.57 and SD 1.12) compared with HT (M = 8.12 and SD: 1.84). The last item which yielded statistically significant results in favour of QT was muscle atrophy, with the HT group showing a higher mean muscle atrophy score (8.13) compared with the QT group (7.34), *p* 0.0298.

All other singular items of the Hacken score did not yield any statistically significant difference between the two graft types.

## 4. Discussion

In our study, the use of QT autograft for ACL reconstruction was associated with better results when compared with STG autograft in terms of overall donor-site morbidity, numbness, size of numbness and muscle atrophy. However, a transient increase in postoperative pain at the 7-day mark might negatively impact the early rehabilitation period.

The use of QT autograft for ACL reconstruction has seen a drastic increase in recent years [16]. This is largely due to the very encouraging scientific data, which have shown how QT has comparable functional outcomes to STG and BPTB autografts, making it a viable alternative. Furthermore, the survival rates amongst these three autografts have been proven to be similar.

In a recent systematic review and meta-analysis conducted by Runer et al. [17], QT autograft was found to have superior functional results when compared with HT and similar when compared with BPTB. However, QT was found to be associated with significantly decreased donor-site pain when compared with BPTB and STG. Similar findings were described by Runer et al. [18] in their 2-year prospective randomised controlled trial, which once again found comparability amongst functional outcomes and lower donor-site morbidity in QT when compared with HT. These results were, however, in contrast with our findings of slightly increased donor-site pain at 7 days postreconstruction in QT patients when compared with HT. Interestingly, Lind et al. also found that QT patients had higher levels of quadriceps muscle deficiency when compared with STG. This was not found in our results where the subjective “quadriceps wasting/atrophy” showed statistically significant results in favour of the QT group. Similar results to ours were also reported by Kunze et al. [19] who found that anterior kneeling pain rates were no different between QT and STG cohorts. However, they did find that STG patients had statistically significant increased incidence of lower leg numbness and irritation when compared with QT patients, potentially due to smaller incision sites—these findings were confirmed by our results.

In a further study conducted by Piussi et al. [20], it was found how there were no statistically significant differences in donor-site morbidity when comparing QT and STG autografts in 2-year patient reported outcomes. More specifically, they showed how no QT patients reported cases of quadriceps bleeding, tenderness, numbness or irritation at the graft harvest site—this was similar to our findings where numbness and irritation were more common findings in patients reconstructed with STG autograft. In addition, they only reported one case of graft rupture at 2 years in a patient reconstructed with STG autograft. There were no cases of graft rupture in our cohorts.

Baranoff et al. [21] compared donor-site morbidity in STG, BPTB and QT. More specifically, they looked at tenderness, numbness, irritation, anterior knee pain and difficulty in kneeling or knee-walking. Tenderness, numbness or irritation at the donor site was present in 4.7% of the patients in the QT group whereas the STG group showed a prevalence of 15.3%. Anterior knee pain and difficulty in kneeling or knee-walking were more common in patients reconstructed with HT autograft. Similarly, our results showed that numbness at the donor site was more common in the HT cohort compared with the QT cohort. On the contrary, our results suggested how there was no statistically significant difference in anterior kneeling pain. Similar findings were reported by Mehran et al. [22] in their systematic review of 55 studies, where anterior knee pain in QT was found in 9.7% of the patients and kneeling pain in 9.5%. These results were comparable with STG and BPTB rates. They concluded by stating how no overall complication in QT was disproportionally higher compared with other graft types.

The similarity in donor-site outcomes between QT and HT was also shown by Hacken et al. [23] in their meta-analysis where they provide evidence in favour of QT and STG compared with BPTB when analysing rates of anterior knee pain and kneeling pain. They concluded by claiming that the ideal graft choice should always be a personalised, patient specific surgical choice.

Moreover, our study also found a potential weak association between low mood (as seen by the PHQ-9 results) and donor-site morbidity, with patients reporting higher PHQ-9 scores displaying slightly inferior donor-site morbidity scores at 6 months. Similar outcomes have already been suggested by Piussi [20] in their systematic review looking at depression and anxiety post ACL reconstruction, showing how low mood is associated with poorer rehabilitation and overall outcomes. Similarly, Baranoff et al. [21] claimed that higher pain catastrophizing was significantly correlated with depressive symptoms in the first 2 weeks post-ACL reconstruction. This finding may find clinical relevance in the presence of patients, which present depressive symptoms in the preoperative period, with the possibility of addressing these prior to the surgical reconstruction to avoid unfavourable donor-site outcomes.

Overall, our findings show how patients receiving ACL reconstruction with QT autograft have statistically significant superior overall donor-site questionnaire outcomes when compared with HT autograft. These findings are in agreement with multiple studies who have shown how QT provides similar biomechanical properties to STG and BPTB, whilst retaining a reduced donor-site morbidity [22].

Our study has some limitations. First, our analyses were conducted in a small sample of patients.

Our sample size was reduced as we had strict inclusion criteria. We intentionally excluded all patients who had also undergone associated repairs or reconstructions (meniscal, posterior cruciate ligament) and patients who were under the age of 16 and only included patients with available follow-up data. Furthermore, the study population included only Caucasians and the results may not be applicable to other ethnicities. However, the present is a pilot analysis. Lastly, the distribution of sex and graft choice was not equal due to surgical preference to offer QT in the male population, reducing the reproducibility of our results. The comparison of both donor-site morbidity and functional outcomes in like-for-like patients with both QT and STG warrants a further and ad-hoc designed prospective study for which data collection is currently in progress.

## 5. Conclusion

ACL reconstruction with all-soft-tissue QT autograft showed overall superior donor-site morbidity outcomes when compared with HT autograft. Statistically significant results were also found in favour of QT when comparing numbness and size of numbness at the donor site and self-perceived muscle atrophy. Female sex and low mood have been found to impact donor-site morbidity negatively although larger samples are necessary to confirm this association. Graft choice in ACL reconstruction should always remain an individualized choice, but QT should be considered an equal alternative to other autologous autografts when comparing donor-site morbidity.

## Figures and Tables

**Table 1:** Characteristics of the study sample.

	Value	Mean ± SD	Range
Sample size	83		
Age (years)		25.8 ± 8.2	18–55
Sex: male	51 (61.4%)		
Sex: female	32 (38.6%)		
BMI (kg/m^2^)		23.9 ± 2.8	18.5–34.6
Smoking: yes	31 (37.3%)		
Smoking: no PHQ-9	52 (62.7%)		
Score		5.2 ± 3.0	1–13
Tegner score		7.7 ± 1.3	5–10
VAS 1		6.2 ± 1.2	3.0–9.0
VAS 7		3.0 ± 1.2	1.0–9.0
VAS 14		0.4 ± 0.6	0.0–2.0
3 months		90.1 ± 4.4	80.2–100.0
6 months		93.6 ± 3.4	86.7–100.0

**Table 2:** Characteristics of the study sample by graft type.

Graft type	Age (years)	BMI (kg/m^2^)	Sex	Tegner score
QT: (46, 55.4%)	23.1 ± 4.8 (range: 18–39)	23.5 ± 2.9 (range: 18.5–34.6)	M: 35 (76.1%), F: 11 (23.9%)	8.0 ± 1.3 (range: 5–10)
HT: (37, 44.6%)	29.2 ± 10.2 (range: 18–55)	24.4 ± 2.8 (range: 20.2–32.9)	M: 16 (43.2%), F: 21 (56.8%)	7.3 ± 1.3 (range: 5–10)	

## Data Availability

The data that support the findings of this study are available from the corresponding author upon reasonable request.
